# *Lactobacillus* amplifies DHAMaR1 conversion to attenuate intestinal ischemia-reperfusion injury via decreasing pyroptosis

**DOI:** 10.3389/fimmu.2025.1712761

**Published:** 2025-12-12

**Authors:** Tong Li, Hongxing Cai, Chenghao Qiu, Jianbo Zhang, Shiji Zhou, Peng Zhu, Wenjing Xian

**Affiliations:** 1 Department of Gastrointestinal Surgery, The Second Affiliated Hospital of Chongqing Medical University, Chongqing, China; 2 Department of Anesthesiology, The First Affiliated Hospital of Chongqing Medical University, Chongqing, China

**Keywords:** docosahexaenoic acid, ischemia/reperfusion injury, *Lactobacillus*, Maresin1, pyroptosis

## Abstract

**Background:**

Maresin1 (MaR1), a specialized pro-resolving mediator derived from docosahexaenoic acid (DHA), exerts potent anti-inflammatory and cytoprotective actions. Its therapeutic relevance and underlying mechanisms in intestinal ischemia–reperfusion (I/R) injury remain to be fully defined.

**Methods:**

A murine I/R model was generated by transient occlusion of the superior mesenteric artery. Retinoic acid receptor-related orphan receptor alpha (RORα) and gasdermin D (GSDMD) knockout mice were used to interrogate signaling pathways. Mice received intraperitoneal MaR1 or dietary supplementation with DHA and/or *Lactobacillus* (Lact). Intestinal injury, inflammation, and pyroptosis were assessed by histology, biochemical markers, and Western blotting. Integrated 16S rRNA sequencing, transcriptomics, and metabolomics characterized microbiota composition, host transcriptional profiles, and metabolic shifts.

**Results:**

MaR1 significantly limited epithelial injury and inhibited pyroptosis through ligand-dependent activation of RORα, effects abolished in *Rorα*^-/-^ and *Gsdmd*^-/-^ mice. DHA supplementation elevated systemic MaR1 levels and reproduced protective effects against I/R damage. Remarkably, combined DHA and *Lactobacillus* supplementation provided synergistic benefits, as *Lactobacillus* enhanced DHA-to-MaR1 conversion. Multi-omics analyses confirmed coordinated modulation of inflammatory networks and microbial metabolic activity.

**Conclusions:**

MaR1 and its precursor DHA confer robust protection against intestinal I/R injury by suppressing RORα-driven pyroptosis. The probiotic-mediated amplification of MaR1 biosynthesis highlights a novel microbiota–lipid mediator crosstalk with translational potential. These findings support combinatorial interventions leveraging host–microbe interactions as a promising therapeutic avenue for ischemic intestinal injury.

## 1 Introduction

Intestinal ischemia/reperfusion (I/R) injury is a life-threatening condition characterized by disruption of the mucosal barrier, dysregulation of tight junction proteins, and excessive release of pro-inflammatory mediators, culminating in systemic inflammatory response syndrome with a high mortality rate (1–3). Current therapeutic strategies remain largely supportive, focusing on surgical revascularization and fluid resuscitation, but fail to address the core pathological triad of I/R injury, epithelial barrier collapse, inflammatory amplification, and endotoxin translocation. Pharmacological interventions targeting single pathways (e.g., antioxidants, anti-IL-6 antibodies) have shown limited clinical efficacy due to the interconnected nature of these mechanisms. This therapeutic gap underscores the critical need for multimodal approaches that simultaneously preserve epithelial integrity, modulate immune responses, and maintain microbial homeostasis.

Pyroptosis represents a distinct form of programmed cell death with a strong pro-inflammatory effect, which is characterized by cellular swelling, rupture, and the release of proinflammatory mediators (4–7). Cellular debris and proinflammatory mediators act as damage-associated molecular patterns (DAMPs) could chemotactically attract inflammatory cells and trigger subsequent inflammatory cascade reactions (8–10). In recent years, cell pyroptosis was considered to be one of the key mechanisms to exacerbates inflammatory response during the heart, liver and kidney I/R injury (11–13). Emerging evidence directly links pyroptosis to intestinal I/R injury, and reverse intestinal morphology and function impairment by decreasing pyroptosis of intestinal epithelial cells (14). Therefore, inhibiting cell pyroptosis is also a potential therapeutic approach to alleviate intestinal I/R injury.

**Scheme 1:**
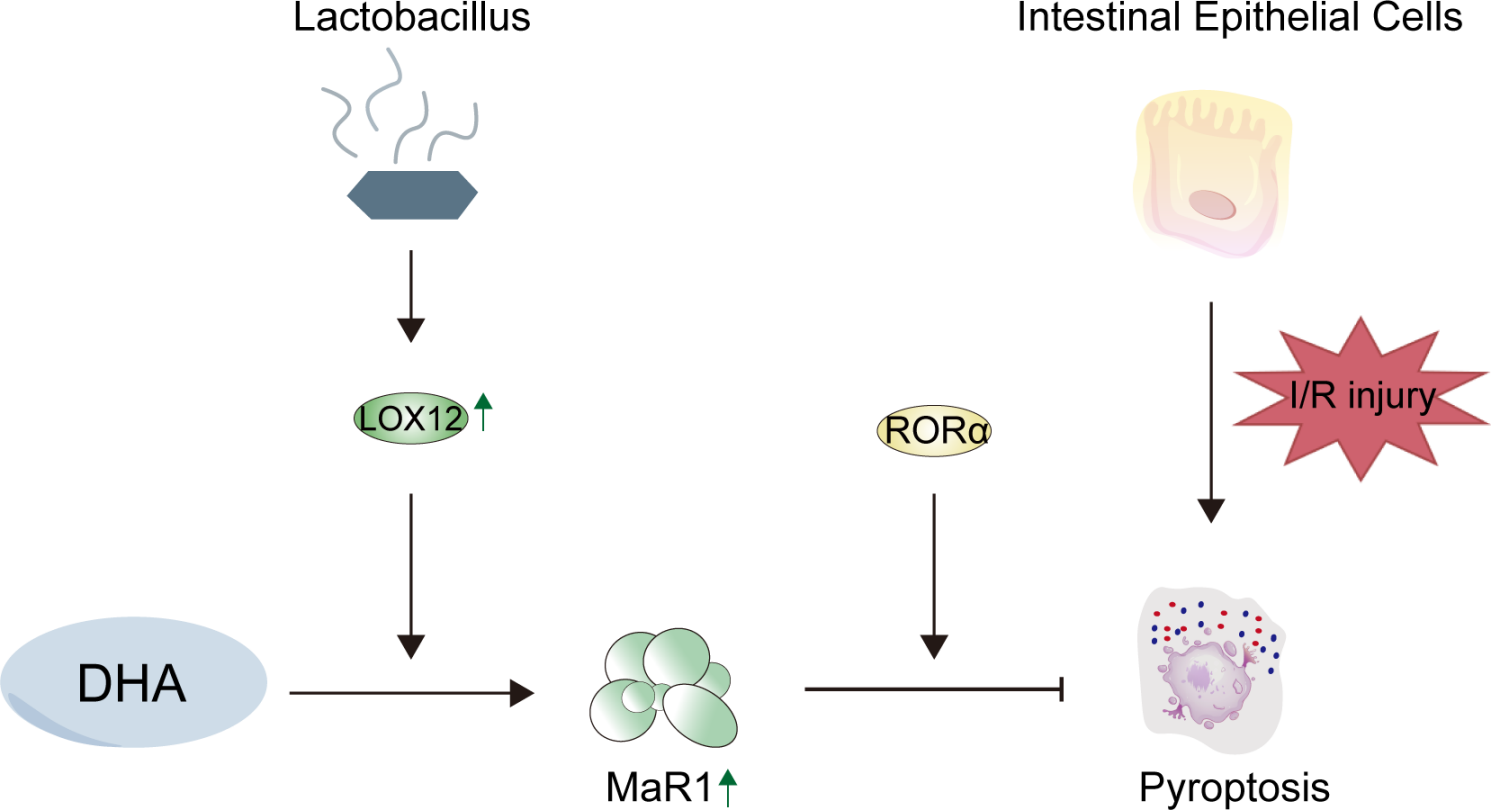
Schematic diagram summarizing the mechanism of Lactobacillus–DHA–MaR1–RORα–pyroptosis interactions.

MaR1 is an emerging pro-resolving lipid mediator that promotes the resolution of inflammation, which is synthesized from docosahexaenoic acid (DHA) via the catalysis of LOX12 (15). Previous studies have shown that MaR1 can promote the polarization of macrophages from the M1 to the M2 phenotype, reduce the release of proinflammatory mediators, and promote tissue repair (16–18). Unlike previous studies focusing on liver I/R injury (19), the present work investigates intestinal epithelial cells and suggests a potential mechanism through which MaR1 synthesis may be regulated by RORα. Our findings indicate that RORα-dependent MaR1 signaling contributes to the suppression of epithelial pyroptosis during I/R injury. In addition, we propose a possible microbiota–lipid mediator interaction, whereby Lactobacillus appears to enhance DHA-to-MaR1 conversion and promote epithelial protection. Collectively, these results point toward a “microbiota–MaR1–RORα–epithelium” axis, providing new insights into the regulatory network of MaR1 in intestinal I/R injury. As the primary physical barrier of the gut, intestinal epithelial cells represent the first line of defense against I/R-induced mucosal injury, yet their capacity for endogenous MaR1-mediated protection remained unexplored. Therefore, we hypothesize that MaR1 may play a similar protective role in the process of intestinal I/R injury. This focus particularly relevant when considering therapeutic interventions like Lactobacillus supplementation, which directly interacts with the intestinal epithelium through pattern recognition receptor (PRR) signaling and metabolite exchange. To provide a conceptual overview, a schematic diagram (**Scheme 1**) summarizes the proposed mechanism of *Lactobacillus*–DHA–MaR1–RORα–pyroptosis interactions, highlighting their integrated roles in preserving epithelial integrity during I/R injury.

In this study, we systematically explored the protective effects of MaR1 and its metabolic precursor DHA on intestinal I/R injury, and we also focus on the underlying molecular mechanisms. These findings not only enhance our understanding of the protective mechanisms of MaR1 cells in the gut, but also underscore the translational potential of combining nutritional and probiotic interventions, offering new therapeutic strategies for patients at risk of intestinal I/R injury.

## 2 Materials and methods

### 2.1 Animals

Male C57BL/6 mice aged 6–8 weeks and weighing 19–23 g were purchased from the Animal Experiment Center of Chongqing Medical University. A total of 15–25 mice were randomly divided into different groups with 3–5 mice per group. The control group did not undergo surgery, while the I/R group received surgical intervention. In the I/R + MaR1 groups, MaR1(Cayman Chemical, Cat# 10878) was administered at doses of 50 ng, 100 ng, or 150 ng after surgery. To investigate the protective effects of *Lactobacillus reuteri* (Lact, BNCC337178) and DHA (Sigma-Aldrich, Cat# D2534) on ischemic intestinal injury, Lact was resuspended in pre-chilled phosphate-buffered saline (PBS) and administered to mice by oral gavage at a dose of 1 × 10^8^ CFU per day. DHA was administered at 300 mg/kg by daily oral gavage for 14 consecutive days.

*Gsdmd*^-/-^ mice were purchased from Shanghai Southern Model Biotechnology Co., Ltd. *Rorα*^-/-^ mice were purchased from Wuhan Mouse Lab Technology Co., Ltd. All animal experiments conducted in this study followed the national ethical principles and standards for animal welfare and were approved by the Ethics Committee of the Second Affiliated Hospital of Chongqing Medical University (approval number: 14 in 2023).

### 2.2 Mouse intestinal ischemia-reperfusion model

The intestinal ischemia-reperfusion model was established based on previous studies (20). All the mice were fasted for 12 hours before surgery and had free access to water. Mice were anesthetized by intraperitoneally injected with amobarbital sodium (Sigma–Aldrich, Cat# PHR8850) at a dosage of 50 mg/kg body weight. The abdomen was opened along the median abdominal line, and the superior mesenteric artery (SMA) was isolated. The SMA was clamped with a microarterial clip to induce intestinal ischemia. Successful vascular occlusion was confirmed by immediate visual verification of diminished intestinal peristaltic activity and characteristic mesenteric pallor. After precisely maintained 45 minutes of ischemia, the arterial clip was removed to allow reperfusion, and the abdomen was closed layer by layer. The mice were euthanized 4 hours later for tissue collection. For MaR1 pretreatment, the required dose of MaR1 was intraperitoneally injected 30 minutes prior to surgery, followed by the induction of intestinal I/R injury.

### 2.3 Cell culture and hypoxia/reoxygenation model

The small intestinal epithelial cell line IEC-6 was purchased from the Shanghai Fuheng Cell Bank. The cells were cultured in Roswell Park Memorial Institute (RPMI) 1640 complete medium supplemented with 10% fetal bovine serum, 1% penicillin and streptomycin and maintained in a humidified atmosphere with 5% CO2 at 37°C. To establish the H/R model, cells were seeded in 6-well plates. Before hypoxia, the conditioned medium was replaced with serum-free medium, and immediately transferred to a hypoxic chamber (94% N2, 5% CO2, and 1% O2) for 12 hours. For reoxygenation, cells were gently rinsed with pre-warmed PBS and replenished with complete growth medium (RPMI-1640 supplemented with 10% fetal bovine serum) before transfer to a standard normoxic incubator (95% air/5% CO_2_) under identical thermal-hygrometric conditions for 2-hour reperfusion.

### 2.4 Determination of intracellular Maresin-1 levels in IEC-6 cells

IEC-6 cells were treated with DHA at graded concentrations of 20 and 100 µg/mL. Following treatment, the cells were washed twice with ice-cold phosphate-buffered saline (PBS) to remove residual medium and extracellular MaR1. For intracellular lipid extraction, the cells were harvested and resuspended in 1 mL of ice-cold methanol: PBS (2:1, v/v), followed by vigorous vortexing for 30 s and brief sonication (2–3 min) on ice. The lysates were then centrifuged at 12,000 × *g* for 10 min at 4°C, and the resulting supernatants containing lipid mediators were collected. The concentration of Maresin-1 (*MaR1*) was quantified using a Maresin-1 ELISA kit (Cayman Chemical, Cat# 501150) according to the manufacturer’s protocol.

### 2.5 Pathohistological examination

Following anesthesia with 50 mg/kg amobarbital sodium (i.p.), mice underwent cervical dislocation. Distal ileal segments (5 cm proximal to the ileocecal junction) were immediately collected and were fixed in 4% paraformaldehyde for 24 hours. Fixed tissues were paraffin-embedded, sectioned at 4 μm thickness, and stained with hematoxylin-eosin (H&E). Histopathological evaluation was performed according to Chiu’s grading criteria (21).

### 2.6 MDA content determination

Malondialdehyde (MDA) quantification was performed using the BC0025 assay kit (Solarbio) according to the manufacturer’s instructions. Briefly, peripheral blood was centrifuged at to obtain serum for analysis. The MDA working solution was prepared by mixing reagent two with reagent one and incubated at 70°C to ensure complete dissolution. The reaction mixture containing the sample, MDA working solution, and reagent three was incubated at 100°C for 60 minutes. After cooling and centrifugation, the absorbance at 532nm and 600nm was measured using a spectrophotometer. MDA content was calculated using the formula provided by the manufacturer.

### 2.7 SOD activity assay

Superoxide dismutase (SOD) activity was assessed using the BC0175 assay kit (Solarbio) following the manufacturer’s protocol. Peripheral blood was centrifuged to obtain clear supernatants for the assay. Reagents were prepared as specified, and the reaction was initiated by the addition of the sample and reagents to a 96-well plate. After a 30-minute incubation at 37°C, the absorbance at 560nm was measured. SOD activity was calculated based on the inhibition percentage and the formula provided by the manufacturer.

### 2.8 GSH content measurement

Glutathione (GSH) levels were determined using the BC1175 assay kit (Solarbio) as per the instructions. Tissue samples were homogenized in reagent one, and the supernatant was collected after centrifugation. A standard curve was prepared by diluting the GSH standard provided. The reaction mixture, containing the sample or standard, reagent two, and reagent three, was incubated at room temperature. The absorbance was read at 412nm, and GSH content was calculated using the standard curve and the formula provided by the manufacturer.

### 2.9 Inflammatory cytokine detection

Serum inflammatory cytokines were quantified using enzyme-linked immunosorbent assay (ELISA). Whole blood samples were centrifuged at 1,500g for 15 min to obtain supernatants. Commercial ELISA kits were employed for detection: IL-1β (Beyotime Biotechnology, Cat# PI301), IL-18 (Beyotime Biotechnology, Cat# PI553), TNF-α (Beyotime Biotechnology, Cat# PT512) and IL-6 (Beyotime Biotechnology, Cat# PI326). The assays were performed following the manufacturer’s protocol, and absorbance at 450 nm was measured using a microplate reader.

### 2.10 Detection of intestinal permeability

Intestinal permeability was measured using 4 kDa fluorescein isothiocyanate-dextran (Sigma–Aldrich, Cat# FD-4). Prior to modeling, the mice were administered FD-4 via gastric injection at a dose of 0.5 mg/g body weight. The terminal ileum was then clamped, and blood was collected after 4 hours of reperfusion. The blood was centrifuged at 4000 r/min to collect the supernatant, which was subsequently placed in a multifunctional microplate reader (thermos, SpectraMax) to measure the fluorescence intensity at 480/520 nm.

### 2.11 Tissue immunofluorescence

The intestinal tissue was fixed with 4% paraformaldehyde, embedded in paraffin, and sectioned. Sections were deparaffinized with xylene and rehydrated through descending ethanol concentrations. Antigen retrieval was performed in 10 mM citrate buffer (pH 6.0) using microwave heating. After blocking with 5% normal goat serum (Beyotime, Cat# C0265) for 1 h at room temperature, sections were incubated with rabbit anti-occludin primary antibody (Proteintech, Cat# 27260-1-AP, 1:1000) overnight at 4°C in a humidified chamber. The next day, after washing with PBS, the sections were incubated with the secondary antibody (Goat Anti-Rabbit IgG, Proteintech, Cat# SA00013-2, 1:1000) at room temperature in the dark for 30 minutes. After washing, the sections were incubated with DAPI at room temperature in the dark for 10 minutes, after which a fluorescence quencher was added to seal the slides. The fluorescence intensity was observed under a fluorescence microscope.

### 2.12 Western blotting

The small intestine was rinsed with PBS, and 20 mg of intestinal tissue was collected. Then, 200 μl of tissue lysis buffer containing protease inhibitors was added, and the mixture was fully lysed in a homogenizer. Subsequently, the lysate was incubated on ice for 30 minutes and centrifuged at 13,000 rpm for 15 minutes. The supernatant was collected, and the protein concentration was determined using a BCA assay kit. One-fourth of the volume of 5X loading buffer was added, and the mixture was heated in a metal bath at 100°C for 10 minutes. Electrophoresis was performed with 30 μg of protein per well. After electrophoresis, the proteins were transferred to a PVDF membrane, which was blocked with 5% nonfat milk in TBST (TBS with 0.1% Tween-20) for 1 hour with gentle agitation. Primary antibodies were diluted in blocking buffer as follows: NLRP3 (Proteintech, Cat# 30109-1-AP, 1:1000),Casp1 (Proteintech, Cat# 81482-1-RR, 1:1000),Cleaved-casp1 (Cell Signaling Technology, Cat# 89332T, 1:1000), GSDMD (Abcam, Cat# ab209845, 1:1000), cleaved-GSDMD (Cell Signaling Technology, Cat# 10137s, 1:1000), and β-actin (Sigma-Aldrich, Cat# A5441, 1:1000). The membrane was then incubated with primary antibodies overnight. The next day, the membrane was washed with TBST and incubated with an HRP-labeled goat anti-rabit secondary antibody (Proteintech, Cat# SA00001-2, 1:2000) at room temperature for 1 hour. Finally, the membrane was developed with enhanced chemiluminescence (ECL) reagent and imaged using a molecular gel imaging system.

### 2.13 16S rRNA sequencing analysis of the gut microbiota

Terminal ileum content (~5 cm proximal to ileocecal junction) was collected: segment exteriorized; lumen optionally flushed with ice-cold PBS; mucosa scraped for adherent content/microbiota. Samples were immediately frozen in liquid nitrogen and stored at -80°C for DNA extraction. DNA was extracted from terminal ileum content samples, and PCR amplification was performed on the V3-V4 variable region of the 16S rRNA gene. The amplified products were subjected to paired-end sequencing on the Illumina PE250 platform (Illumina, San Diego, USA). To ensure the quality of the sequencing data, fastp software (version v0.19.6, obtained from https://github.com/OpenGene/fastp) was used for sequence quality control, followed by sequence stitching using FLASH software (version v1.2.11, sourced from the https://sourceforge.net/projects/flashpage/files/flash-1.2.11.tar.gz/download). The optimized sequences were denoised using the DADA2 plugin within the QIIME2 pipeline based on default parameters. For species taxonomy analysis of the denoised sequences, we utilized the SILVA 16S rRNA gene database (version v138) and the naive Bayes classifier within QIIME2 to classify the ASVs. All the data analyses were conducted on the Majorbio Cloud Platform (https://cloud.majorbio.com).

### 2.14 Metabolomic detection and analysis

The UHPLC-Q Exactive HF-X system (Thermo Fisher Scientific) was used for LC–MS/MS analysis of the samples. The resulting data were processed using the metabolomics software Progenesis QI (provided by Waters Corporation, Milford, USA). Simultaneously, MS and MS/MS spectral information was matched with the public metabolic databases HMDB (v5.0, http://www.hmdb.ca/) and Metlin (v2.0, https://metlin.scripps.edu/) to obtain metabolite details. All the data analyses were also performed on the Majorbio Cloud Platform (https://cloud.majorbio.com).

### 2.15 Transcriptomic detection and analysis

Total RNA was extracted from intestinal tissues, after which the concentration, purity, and integrity of the extracted RNA were rigorously tested. Subsequently, cDNA was synthesized through reverse transcription using mRNA as a template, after which a DNA library was constructed. Sequencing was performed on the Illumina NovaSeq 6000 platform. To ensure the quality of the sequencing data, fastp software (version v0.19.6, https://github.com/OpenGene/fastp) was used to filter all sequencing reads, which produced high-quality sequencing data. Finally, the filtered data were uploaded to the Majorbio Cloud Platform (https://cloud.majorbio.com) for clustering, KEGG, and other related analyses.

### 2.16 Statistical analysis

Data were statistically analyzed using SPSS 25.0 software. The data are expressed as the mean ± standard deviation (mean ± SD). One-way ANOVA was used to analyze differences among different groups, and the LSD test was used for comparisons between two groups. The level of significance was set at α=0.05. P < 0.05 was considered to indicate statistical significance.

## 3 Results

### 3.1 MaR1 alleviated intestinal I/R injury in mice

**Figure 1:**
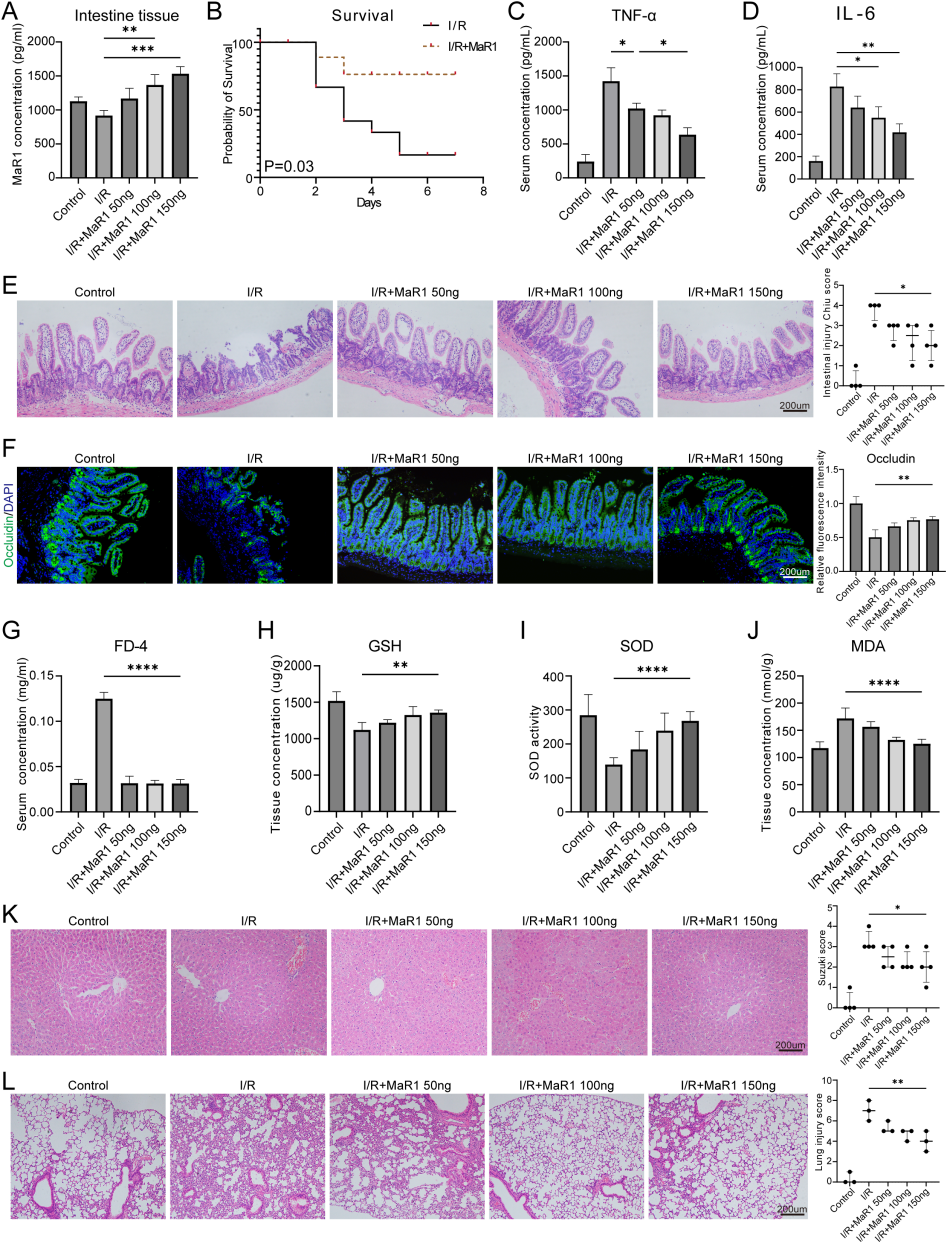
MaR1 alleviated intestinal I/R injury in mice **(A)** The MaR1 concentration in intestinal tissue was quantified by ELISA. **(B)** Survival statistics of intestinal I/R injury mice treated with or without MaR1. **(C)** Serum TNFα and **(D)** IL-6 concentration detected by ELISA. **(E)** The histopathological damage of I/R injury was estimated by H&E staining and the Chiu’s score. **(F)** The expression of Occludin in intestinal tissues was detected by immunofluorescence. **(G)** Intestinal permeability test determined by FITC-dextran transepithelial permeability. Detection of antioxidant capacity of intestinal tissue, including **(H)** GSH level, **(I)** SOD activity, and **(J)** MDA concentration. **(K)** The histopathological damage of I/R injury was estimated by H&E staining and the liver injury score. **(L)** The histopathological damage of I/R injury was estimated by H&E staining and the lung injury score. Data are presented as mean ± SEM. *P < 0.05, **P < 0.01, ***P < 0.001, and ****P < 0.0001.

Mice were pretreated with different concentrations of MaR1 and then subjected to intestinal I/R injury. MaR1 supplementation elevated intestinal MaR1 levels in a dose-dependent manner while suppressing ischemia/reperfusion (I/R)-induced upregulation of pro-inflammatory cytokines (TNF-α, IL-6) (**Figures 1A, C, D**). MaR1 treatment significantly prolonged survival duration and reduced mortality risk in mice with intestinal I/R injury (*P* = 0.03) (**Figure 1B**). MaR1-treated mice exhibited reduction in intestinal mucosal desquamation (**Figure 1E**), accompanied by upregulation of Occludin expression in epithelial cells (**Figure 1F**), collectively enhancing mucosal barrier integrity as evidenced by decrease in FD-4 permeability (**Figure 1G**). In addition, MaR1 supplementation increased the synthesis of the antioxidant-GSH (**Figure 1H**) and SOD (**Figure 1I**), and reduced the production of the lipid peroxide-MDA (**Figure 1J**). Moreover, MaR1 decreased tissue damage in liver and lung induced by I/R (**Figures 1K, L**). These findings suggest MaR1 exerts powerful tissue-protective effects during small intestinal I/R injury.

### 3.2 MaR1 attenuates intestinal I/R injury by reducing cell pyroptosis

**Figure 2:**
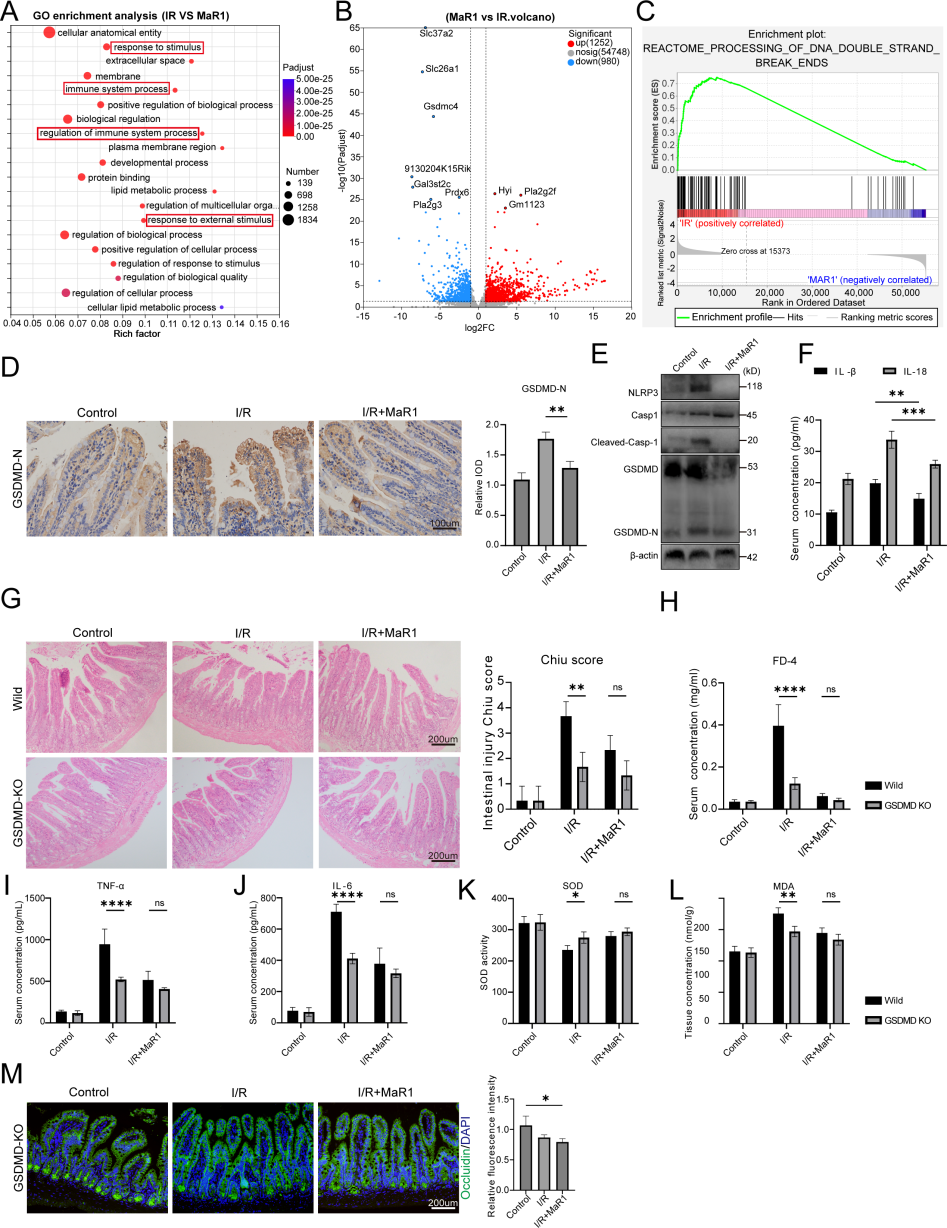
MaR1 attenuates intestinal I/R injury by reducing cell pyroptosis. **(A)** Gene ontology (GO) pathway enrichment analyses of the upregulated DEGs between I/R and I/R+MaR1 groups (FDR < 0.05). **(B)** Volcano plots comparing the DEGs between I/R and I/R+MaR1 groups. **(C)** Enrichment analysis of “DNA double-strand break ends” pathway. **(D)** Representative immunohistochemistry images of GSDMD-N expression and quantification of the area of GSDMD-N immunohistochemical staining. **(E)** NLRP3, Caspas-1 and GSDMD expression in intestinal tissue were detected by western blotting. **(F)** Serum IL-1β and IL-18 concentration detected by ELISA. **(G)** The histopathological damage of I/R injury was estimated by H&E staining and the Chiu’s score in GSDMD-KO mice, BM: Bowel Mucosa, Vill: Villi. **(H)** Intestinal permeability test determined by FITC-dextran transepithelial permeability. **(I, J)** Serum TNFα and IL-6 concentration detected by ELISA. **(K, L)** Detection of antioxidant capacity of intestinal tissue, including SOD activity and MDA concentration. **(M)** The expression of Occludin in intestinal tissues from GSDMD-KO mice was detected by immunofluorescence. Data are presented as mean ± SEM. *P < 0.05, **P < 0.01, ***P < 0.001, and ****P < 0.0001.

To investigate the mechanism by which MaR1 alleviates intestinal I/R injury, we performed transcriptomic analysis of the intestine. Through transcriptome analysis of mouse intestines, we discovered that MaR1 exerts tissue protective effects by modulating pathways such as “response to stimulus”, “immune system process”, and “positive regulation of biological processes” (**Figures 2A, B**). Furthermore, we observed that MaR1 significantly upregulated the expression of genes associated with the “processing of DNA double-strand break ends” pathway (**Figure 2C**), which reflects the organism’s repair function in response to cellular damage. Subsequently, we investigated the impact of MaR1 on cell pyroptosis. Immunohistochemical (IHC) quantification revealed substantial accumulation of gasdermin D (GSDMD) pore-forming proteins in intestinal epithelial cells of I/R-injured mice, while immunoblotting detected elevated levels of its active N-terminal fragment (GSDMD-N) (**Figures 2D, E**). Concomitantly, serum concentrations of pyroptosis-driven cytokines IL-1β and IL-18 increased respectively compared to sham controls (**Figure 2F**). Strikingly, treatment with MaR1 effectively reduced the expression of GSDMD and GSDMD-N maturation, concomitantly reducing systemic IL-1β/IL-18 levels (**Figures 2D-F**). The above results indicated MaR1 effectively reduced cellular pyroptosis during intestinal I/R injury.

To further validate the contribution of pyroptosis inhibition to MaR1-mediated protection, we utilized GSDMD-knockout (GSDMD-KO) mice. Compared with wild-type mice, GSDMD-KO mice exhibited significantly reduced mucosal damage (**Figure 2G**), prevention of I/R-induced intestinal hyperpermeability (**Figure 2H**), diminished proinflammatory cytokine responses (TNF-α, IL-6; **Figures 2I, J**), and enhanced antioxidant capacity (**Figures 2K, L**). Notably, immunofluorescence analysis revealed that while I/R injury markedly downregulated Occludin expression in intestinal epithelial cells, MaR1 treatment failed to restore this tight junction protein in GSDMD-KO mice (**Figure 2M**). This finding provides direct evidence that GSDMD is indispensable for the epithelial barrier–protective effects of MaR1, firmly establishing pyroptosis inhibition as a core mechanism underlying its intestinal protection.

### 3.3 MaR1 reduces pyroptosis by activating RORα

**Figure 3:**
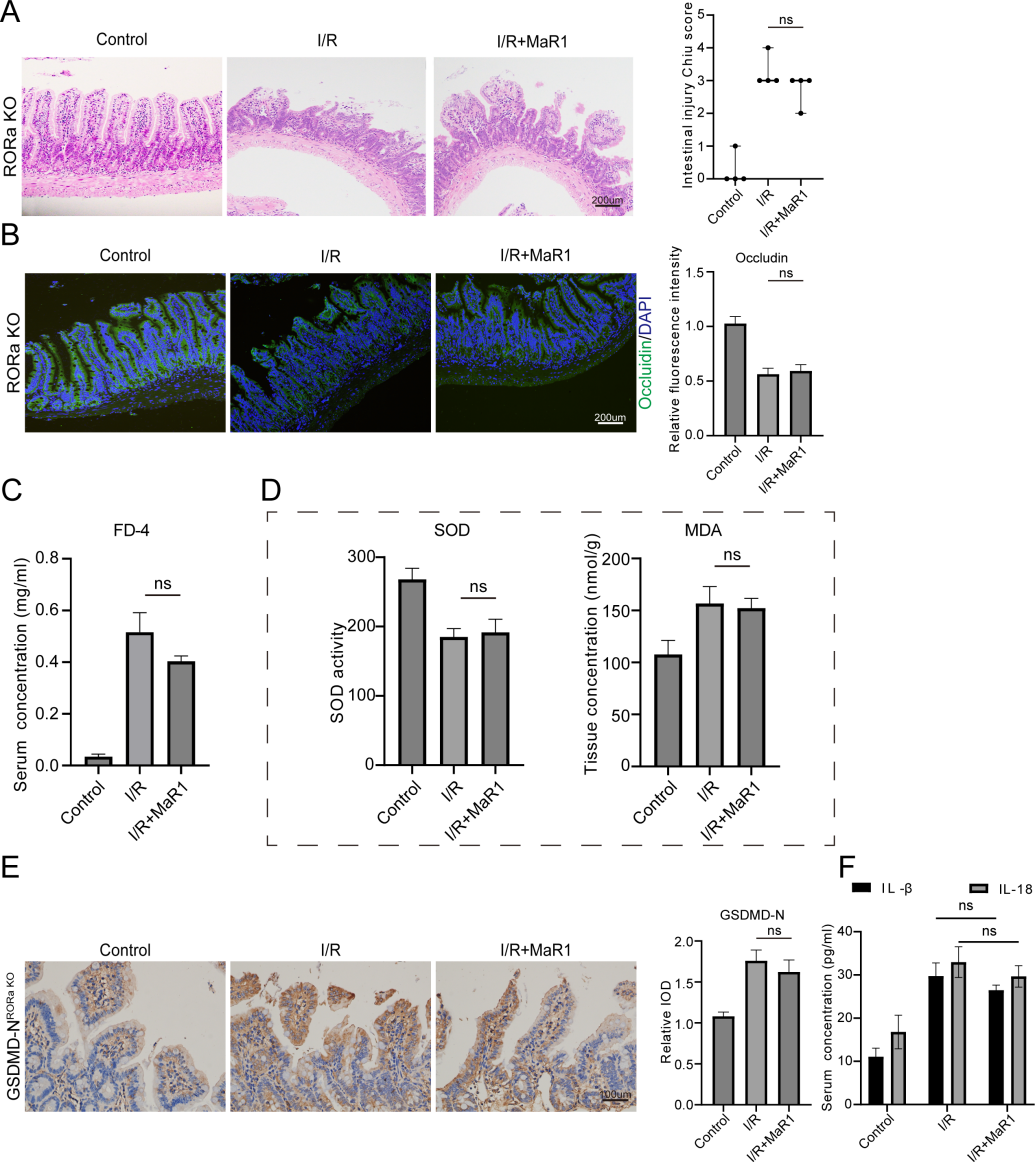
MaR1 reduces pyroptosis by activating RORα. **(A)** The histopathological damage of I/R injury was estimated by H&E staining and the Chiu’s score in RORα-KO mice. **(B)** The expression of Occludin in intestinal tissues from RORα-KO mice was detected by immunofluorescence. **(C)** Intestinal permeability test determined by FITC-dextran transepithelial permeability. **(D)** Detection of antioxidant capacity of intestinal tissue, including SOD activity and MDA concentration. **(E)** Representative immunohistochemistry images of GSDMD-N expression and quantification of the area of GSDMD-N immunohistochemical staining. **(F)** Serum IL-1β and IL-18 concentration detected by ELISA. Data are presented as mean ± SEM.

RORα serves as a nuclear receptor for MaR1, with MaR1 not only upregulating RORα expression but also enhancing its biological activity (15). Our previous studies have demonstrated that MaR1 can reduce macrophage pyroptosis by activating RORα during liver I/R injury (19).To investigate whether this pathway mediates protection in intestinal I/R, we subjected RORα-knockout (KO) mice to intestinal I/R with or without MaR1 treatment. Critically, administration of MaR1 in RORα-KO mice failed to improve intestinal mucosal damage or barrier dysfunction (**Figures 3A, B**), did not reduce elevated intestinal permeability (**Figure 3C**), and was unable to restore antioxidant defenses as evidenced by unaltered SOD activity and MDA content (**Figure 3D**). Furthermore, MaR1 treatment showed no suppressive effect on pyroptosis signaling in these mice, with GSDMD-N cleavage levels remaining unchanged (**Figure 3E**) and plasma IL-1β/IL-18 concentrations unaffected (**Figure 3F**). These findings collectively demonstrate that RORα activation is essential for MaR1’s protective effects against intestinal I/R injury, acting through suppression of the pyroptotic cascade.

### 3.4 Supplementation with DHA supplementation enhances the MaR1 biosynthesis and reduces tissue damage

**Figure 4:**
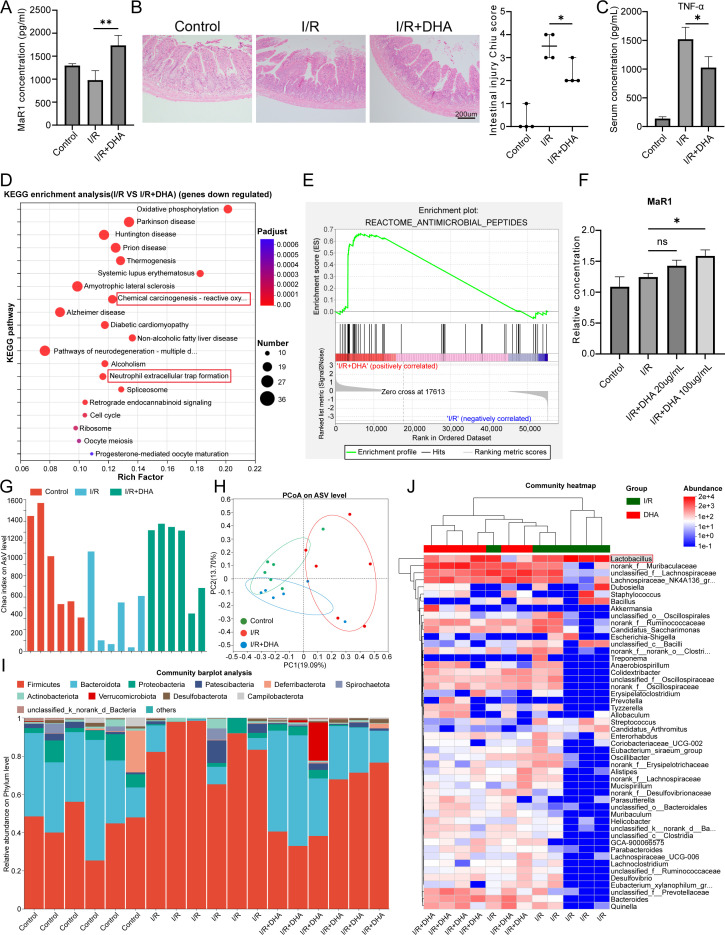
DHA supplementation enhances MaR1 Biosynthesis and reduces tissue damage. **(A)** Serum MaR1 concentration detected by ELISA. **(B)** H&E staining and the Chiu’s score in intestinal I/R injury mice treated with or without DHA. **(C)** Serum TNFα concentration detected by ELISA. **(D)** KEGG enrichment analysis of downregulated genes in the I/R Group *vs* I/R+DHA Group (FDR < 0.05). **(E)** Enrichment analysis of the “reactome antimicrobial peptides” pathway. **(F)** Intracellular MaR1 concentration detected by ELISA in IEC-6 cells treated with different doses of DHA. **(G)** Alpha diversity (Chao index) of gut microbiota. **(H)** Principal coordinate analysis (PCoA) based on Bray–Curtis distances at the ASV level showing differences in microbial community composition among groups. **(I)** Community barplot analysis. **(J)** Community heatmap. Data are presented as mean ± SEM. *P < 0.05, **P < 0.01.

DHA serves as a precursor of MaR1, we subsequently investigated the therapeutic potential of dietary DHA supplementation in murine intestinal I/R injury in mice. After 2 weeks of dietary supplementation, serum MaR1 concentrations were significantly elevated (**Figure 4A**), and attenuated intestinal mucosal damage (**Figure 4B**). Furthermore, DHA supplementation attenuates I/R-induced upregulation of pro-inflammatory cytokine TNF-α in serum (**Figure 4C**). Transcriptomic profiling of intestinal tissue revealed that DHA supplementation exerted dual regulatory effects on gene expression profiles, simultaneously, downregulating pro-inflammatory pathways and upregulating the expression of genes associated with “antimicrobial peptides” (**Figures 4D, E**). Intriguingly, *in vitro* experiments with intestinal epithelial cells (IEC-6) revealed a dose-dependent increase in intracellular MaR1 levels following DHA treatment (**Figure 4F**). These results indicate that intestinal epithelial cells may synthesize MaR1 by taking up DHA, contrary to the previous belief that MaR1 is synthesized only in macrophages and platelets (22–24).

Furthermore, we investigated the effects of DHA supplementation on the gut microbiota composition in mice. Intestinal I/R injury significantly reduced gut microbial diversity, while DHA supplementation effectively restored it (**Figures 4G, H**). This restoration was further supported by Shannon index analysis (**Supplementary Figure 1**) and PERMANOVA analysis (**Supplementary Table S1**). High-throughput sequencing produced approximately 26–46 million bases per sample, ensuring sufficient sequencing depth for a precise assessment of microbial diversity (**Supplementary Table S2**). At the phylum level, DHA supplementation significantly altered the microbial composition, increasing the relative abundance of Bacteroidetes while decreasing that of Firmicutes (**Figure 4I**). At the genus level, DHA treatment increased the abundance of *Prevotella*, *Bacteroides*, and *Lachnospira*, while reducing Lactobacillus levels compared with I/R controls (**Figure 4J**).

In addition, KEGG enrichment analysis (**Supplementary Figure 2A**) revealed that differential metabolites were mainly involved in the *NF-κB signaling pathway* and *Th1/Th2 cell differentiation*, indicating activation of inflammation-related metabolic pathways following I/R injury. Consistently, several typical pro-inflammatory metabolites, including *PGE_2_*, *PGF_1_α*, and *LysoPC*, were markedly elevated in the I/R group but attenuated after DHA treatment (**Supplementary Figure 2B**). These findings suggest that DHA supplementation reduces the production of metabolites associated with inflammatory pathways, in agreement with transcriptomic results, thereby highlighting the systemic anti-inflammatory effects mediated by *DHA*-induced gut microbiota remodeling.

### 3.5 *Lactobacillus* enhanced the protective effects of DHA to MaR1

**Figure 5:**
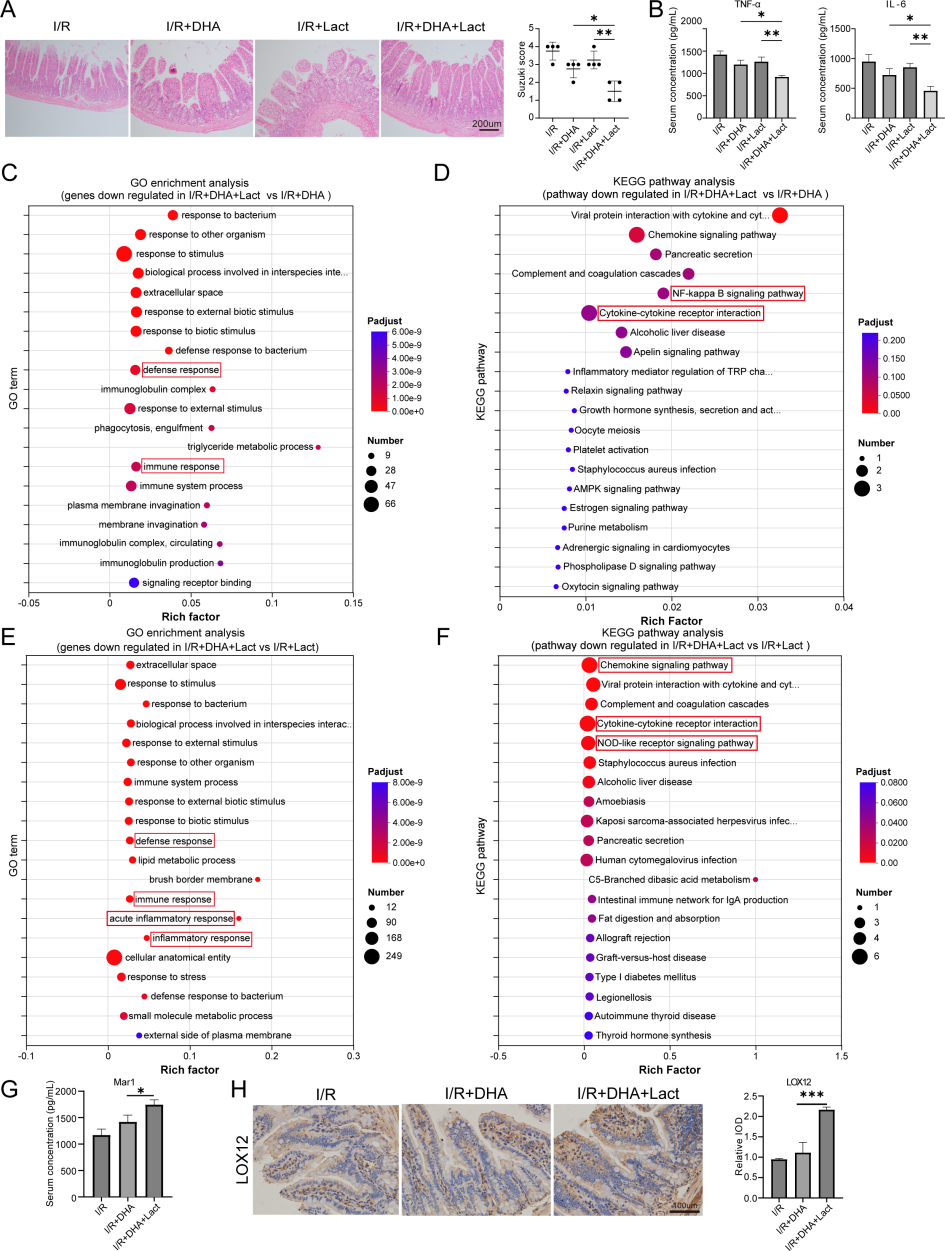
*Lactobacillus* enhanced the protective effects of DHA. **(A)** H&E staining and the Chiu’s score in intestinal I/R injury mice treated with DHA supplementation alone, *Lactobacillus* (Lact) supplementation alone, or combined DHA and Lact supplementation. **(B)** Serum TNFα and IL-6 concentration detected by ELISA. **(C)** GO enrichment analysis of downregulated genes in the I/R+DHA+Lact Group *vs* I/R+DHA Group (FDR < 0.05). **(D)** KEGG pathway analysis between I/R+DHA+Lact Group and I/R+DHA Group (FDR < 0.05). **(E)** GO enrichment analysis of downregulated genes in the I/R+DHA+Lact Group *vs* I/R+Lact Group. **(F)** KEGG pathway analysis between I/R+DHA+Lact Group and I/R+ Lact Group **(G)** Serum MaR1 concentration detected by ELISA. **(H)** Representative immunohistochemistry images of LOX12 expression and quantification of the area of LOX12 immunohistochemical staining. Data are presented as mean ± SEM. *P < 0.05, **P < 0.01, and ***P < 0.001.

Given the shared mechanisms of action between *Lactobacillus* and DHA in anti-inflammatory and antioxidant properties, we subsequently embarked on an investigation to determine whether *Lactobacillus* supplementation could amplify the protective effects of DHA. Mice were divided into four groups: I/R injury, DHA supplementation alone, *Lactobacillus* supplementation alone, and combined DHA and *Lactobacillus* supplementation. Strikingly, the combined treatment group demonstrated markedly superior protection compared to either intervention alone, exhibiting the maximum reduction in histological damage scores and pro-inflammatory cytokine (TNF-α and IL-6) in serum (**Figures 5A, B**). Transcriptomic analysis revealed that combined supplementation with DHA and *Lactobacillus* markedly alleviated intestinal inflammation compared with either treatment alone. Compared with the DHA group, GO enrichment analysis indicated a downregulation of genes associated with defense and immune responses in the DHA+Lact group (**Figure 5C**); the complete list of differentially expressed genes (DEGs) and detailed GO enrichment statistics are provided in **Supplementary Table S3** for reference. KEGG pathway analysis further showed significant suppression of inflammation-related pathways, including the NF-κB signaling pathway and cytokine–cytokine receptor interaction (**Figure 5D**); specific pathway enrichment scores and corresponding DEGs are detailed in **Supplementary Table S4**. This was accompanied by reduced expression of inflammation-related genes such as *Cxcl1*, *Ccl21d*, and *Il22ra1*, with quantitative expression data of these genes presented in **Supplementary Figure 3A**. Similarly, compared with the *Lactobacillus* group, the DHA+Lact combination decreased the expression of genes involved in immune response, acute inflammatory response, and inflammatory response (**Figure 5E**); comprehensive DEG lists and GO enrichment parameters are available in **Supplementary Table S5**. Consistently, KEGG analysis demonstrated downregulation of the chemokine signaling pathway, cytokine–cytokine receptor interaction, and NOD-like receptor signaling pathway (**Figure 5F**); detailed pathway information and DEG statistics are provided in **Supplementary Table S6**. Correspondingly, the expression levels of *Cxcl1*, *Cxcl2*, *Il36g*, and *Defa28* were reduced, with quantitative validation results shown in **Supplementary Figure 3B**.

Furthermore, we discovered that *Lactobacillus* supplementation facilitated the conversion of DHA into MaR1 (**Figure 5G**). Specifically, *Lactobacillus* administration elevated the expression of LOX12 (**Figure 5H**), which is rate-limiting enzymes for the conversion of DHA into MaR1. The strong correlation between elevated MaR1 concentrations and tissue protection suggests this metabolic conversion represents a crucial mechanism underlying the observed synergy. These results establish a novel probiotic-pharmacologic therapeutic strategy in which *Lactobacillus* potentiates the bioconversion of dietary DHA into bioactive MaR1, thereby amplifying tissue protection during intestinal I/R injury.

## 4 Discussion

Our study demonstrates that MaR1 attenuates intestinal I/R injury through RORα-mediated suppression of pyroptosis in intestinal epithelial cells. Importantly, we identified a novel probiotic-nutritional synergy wherein DHA supplementation elevated systemic MaR1 levels, and concurrent *Lactobacillus* administration enhanced this effect by upregulating the expression of LOX12, the rate-limiting enzyme in MaR1 biosynthesis. This synergistic combination achieved greater tissue protection compared to monotherapies, establishing a new therapeutic paradigm for intestinal I/R injury.

MaR1 potently regulates the development of inflammation, Chiang et al. (25) reported that MaR1 can promote phagocytic and efferocytosis functions in macrophages by activating LGR6 while suppressing the release of inflammatory mediators. Jin et al. (17) reported that MaR1 ameliorates rheumatoid arthritis progression by improving the Treg/Th17 imbalance. Han et al. (15) discovered that MaR1 attenuates the progression of nonalcoholic fatty liver disease by promoting the polarization of liver macrophages toward the M2 phenotype. Our previous studies have shown that MaR1 effectively mitigates mitochondrial damage in macrophages and reduces the release of inflammatory cytokines such as IL-1 and TNFα during ischemia–reperfusion (I/R) injury in the brain and liver (19, 26, 27). Consistent with previous reports in macrophages and hepatocytes, our findings confirm that MaR1 exerts protection primarily through pyroptosis inhibition.

This study further underscores the pivotal role of MaR1 in mitigating inflammatory injury. Unlike previous studies that mainly emphasized its actions in inflammatory cells (8, 28), we identify intestinal epithelial cells as a direct and essential target of MaR1 in the context of intestinal I/R injury. Specifically, MaR1 suppresses RORα-dependent epithelial pyroptosis, thereby maintaining mucosal barrier integrity. In contrast to our prior liver I/R work (19), which focused on macrophage-mediated inflammatory resolution, these findings reveal an epithelial-centered protective mechanism unique to the intestine. Moreover, we uncover a microbiota–lipid mediator interaction in which Lactobacillus enhances DHA-to-MaR1 conversion and amplifies this epithelial cytoprotective pathway. Together, these results define a distinct “microbiota–MaR1–RORα–epithelium” axis that integrates microbial metabolism with pro-resolving lipid signaling to maintain intestinal integrity during I/R injury.

Previous studies have suggested that MaR1 is synthesized primarily in platelets and macrophages using DHA as a substrate (29–33). In this study, we report for the first time that small intestinal mucosal cells may possess the capability to synthesize MaR1. Although we solely observed that supplementation with DHA elevated the intracellular (rather than the extracellular in the culture medium) concentration of MaR1. This may be attributed to the relatively low levels of MaR1 synthesis and secretion. Interestingly, we found that DHA supplementation may lead to a decrease in the abundance of *Lactobacillus* in the small intestine, possibly due to the selective modulation of gut microbial composition by DHA, which reduces certain endogenous *Lactobacillus* species. However, exogenous supplementation with specific strains (such as *Lactobacillus reuteri*) may exhibit a stronger capacity for DHA metabolism or promote MaR1 production more effectively. Consistently, *Lactobacillus* supplementation promoted MaR1 synthesis by upregulating the expression of LOX12.

In addition to these changes, DHA supplementation also reshaped the overall gut microbiota composition, characterized by an increased abundance of Bacteroidetes and decreased Firmicutes, accompanied by enrichment of Prevotella and Bacteroides. These taxa have been frequently associated with anti-inflammatory and barrier-protective functions, as members of Bacteroides and Prevotella are known to produce short-chain fatty acids (SCFAs) and other metabolites that modulate host immunity and epithelial integrity (34, 35). The shift toward a Bacteroidetes-dominant profile may therefore contribute to the observed attenuation of inflammatory responses and enhanced mucosal protection. Whether these microbial alterations directly participate in the protective effects or act synergistically with the MaR1–RORα pathway warrants further investigation.

The above results suggest that concurrent supplementation of DHA and *Lactobacillus* could exerts a synergistic protective effect. This combination approach offers exceptional translational potential due to the established safety profiles and widespread availability of both DHA (a well-tolerated ω-3 fatty acid) and *Lactobacillus* (a GRAS-certified probiotic). The superior protection against I/R injury achieved by co-supplementation, compared to individual treatments, underscores its clinical value, particularly for at-risk populations such as surgical patients, the elderly, and critically ill individuals. Importantly, this nutritional strategy avoids the side effects associated with pharmacological interventions while potentially conferring systemic benefits beyond intestinal protection, making it ideally suited for preventive health management and adjunctive therapy in ischemia-prone conditions.

While this study demonstrates that DHA and *Lactobacillus* confer protection against intestinal I/R injury primarily through MaR1, several limitations should be acknowledged. First, DHA serves as a metabolic precursor can be converted into other pro-resolving mediators such as Resolvins and Protectins, and it exerts tissue-protective effects through various pathways. In this study, we specifically focused on the MaR1-RORα-pyroptosis axis and thus cannot exclude potential contributions from other DHA-derived metabolites. Similarly, *Lactobacillus* also exerts tissue-protective effects through multiple mechanisms, which were not investigated in the present study. Additionally, while we highlight the therapeutic potential of this mechanism for intestinal I/R injury, both DHA and *Lactobacillus* have demonstrated efficacy in other pathological contexts, such as inflammatory bowel disease, sepsis, and metabolic disorders. Thus, future studies should explore whether the observed synergy extends to these conditions or is unique to ischemic gut injury.

This study establishes that MaR1 exerts tissue-protective effects by mitigating pyroptosis through the activation of RORα in small intestinal I/R injury. The nutritional-probiotic combination described here offers a clinically feasible approach to prevent I/R injury, particularly valuable for surgical/transplant settings where ischemic insults are predictable. Future studies should explore optimized dosing regimens and their impact on postoperative outcomes.

## Data Availability

The raw sequencing reads are available in the NCBI SRA under BioProject accession PRJNA1367799 (https://www.ncbi.nlm.nih.gov/bioproject/PRJNA1367799).
