# Correction: *Lactobacillus* amplifies DHAMaR1 conversion to attenuate intestinal ischemia-reperfusion injury via decreasing pyroptosis

**DOI:** 10.3389/fimmu.2025.1769659

**Published:** 2026-01-06

**Authors:** Tong Li, Hongxing Cai, Chenghao Qiu, Jianbo Zhang, Shiji Zhou, Peng Zhu, Wenjing Xian

**Affiliations:** 1 Department of Gastrointestinal Surgery, The Second Affiliated Hospital of Chongqing Medical University, Chongqing, China; 2 Department of Anesthesiology, The First Affiliated Hospital of Chongqing Medical University, Chongqing, China

**Keywords:** docosahexaenoic acid, ischemia/reperfusion injury, *Lactobacillus*, Maresin1, pyroptosis

The order of authors in the author list of the published paper was erroneously given as:

“Tong Li, Hongxing Cai, Chenghao Qiu, Jianbo Zhang, Shiji Zhou, Wenjing Xian, Peng Zhu”

The correct author list reads:

“Tong Li, Hongxing Cai, Chenghao Qiu, Jianbo Zhang, Shiji Zhou, Peng Zhu, Wenjing Xian”

The original version of this article has been updated.

