# Supplementary material for: Lactobacillus amplifies DHAMaR1 conversion to attenuate intestinal ischemia-reperfusion injury via decreasing pyroptosis
Source: Front Immunol. 2025 Dec 12;16:1712761. doi: 10.3389/fimmu.2025.1712761 (PMC12741079; doi:10.3389/fimmu.2025.1712761)
Supplement: Supplementary file 1 [file DataSheet1.pdf]

Supplementary Material

1.1 Supplementary Figures

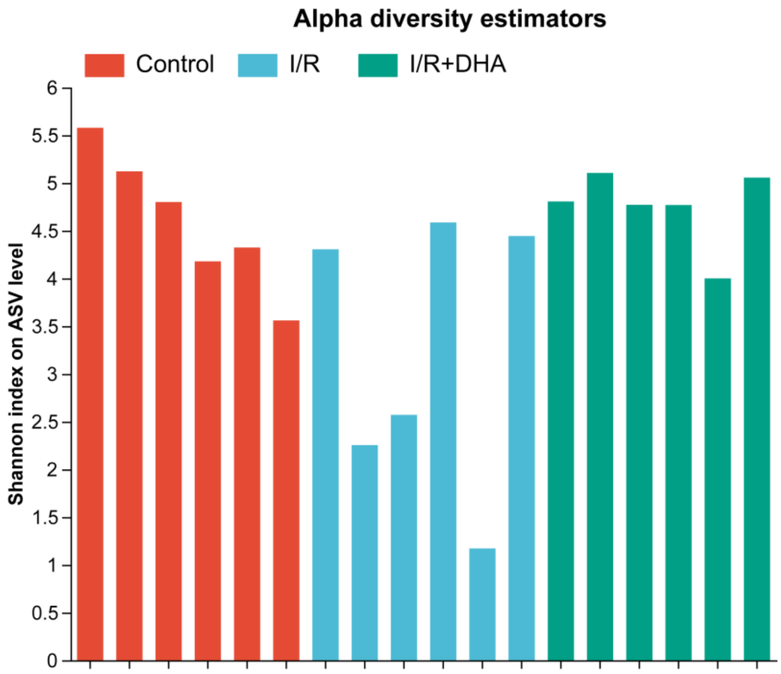

Supplementary Figure 1. Alpha diversity (Shannon index) of gut microbiota. Shannon index of gut microbiota across groups. Each group (n = 6) showed sufficient sequencing depth (2.6–4.6 × 10^7 bases).

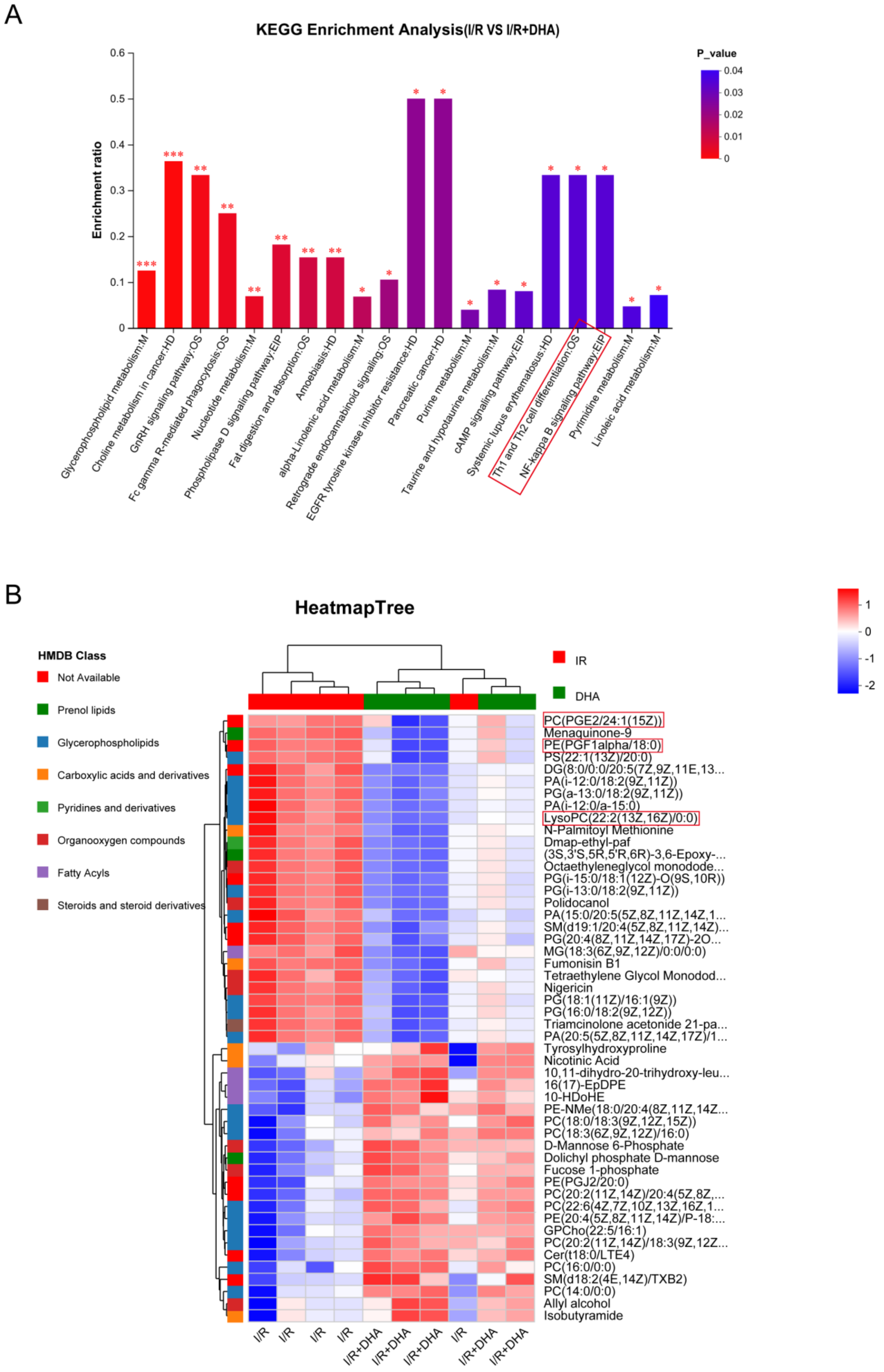

Supplementary Figure 2. Metabolic KEGG enrichment and metabolite clustering analyses between I/R and I/R+DHA groups.

(A) Differential metabolites were significantly enriched in inflammation-related pathways (FDR < 0.05).
(B) HeatmapTree illustrating overall metabolic differences between groups.

A

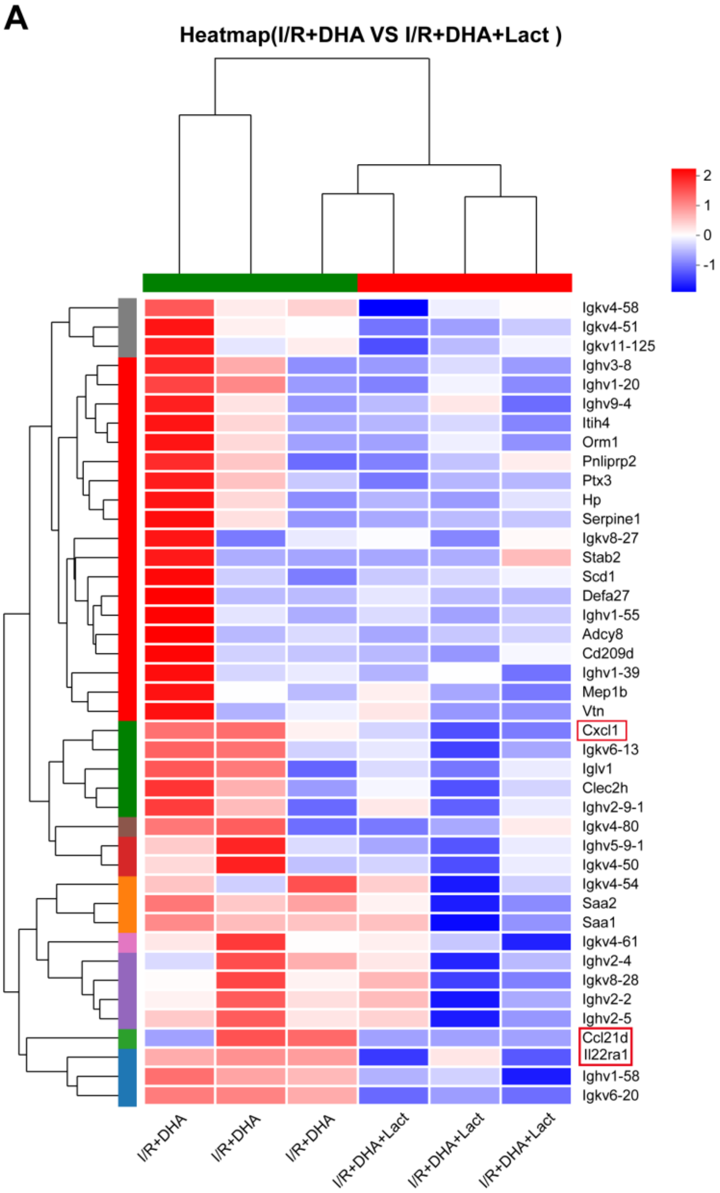

B

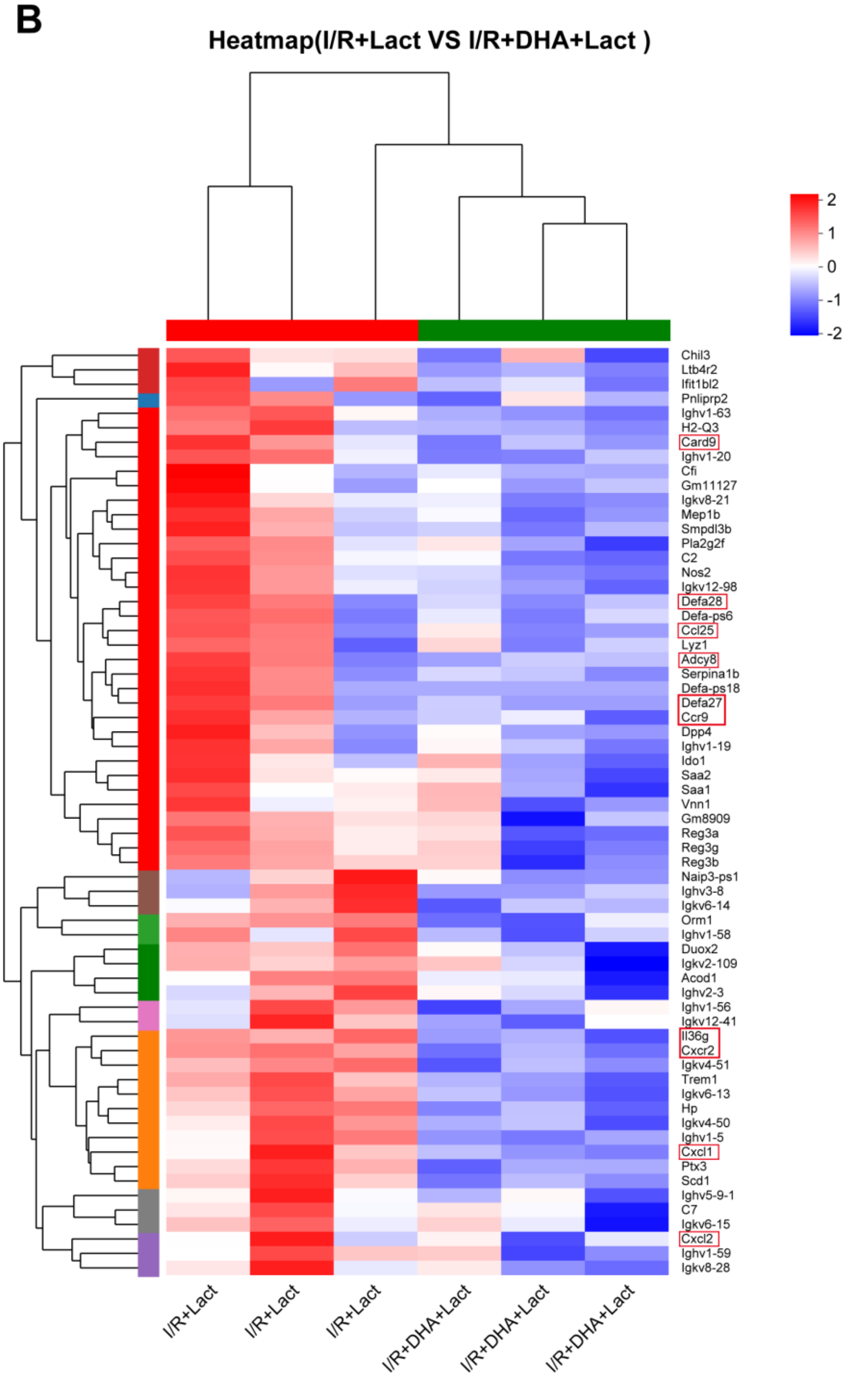

Supplementary Figure 3. Heatmap illustrating differential gene expression profiles

(A) Heatmap showing differential gene expression between the I/R+DHA and I/R+DHA+Lact groups, illustrating distinct clustering patterns and highlighting inflammation-related genes.

(B) Heatmap showing differential gene expression between the I/R+Lact and I/R+DHA+Lact groups, illustrating distinct clustering patterns and highlighting inflammation-related genes.
